# Three-dimensional mapping of distal humerus fracture

**DOI:** 10.1186/s13018-021-02691-0

**Published:** 2021-09-03

**Authors:** Chao Wang, Yong Zhu, Haitao Long, Zhangyuan Lin, Ruibo Zhao, Buhua Sun, Shushan Zhao, Liang Cheng

**Affiliations:** Department of Orthopedics, Xiangya Hospital, Central South University, Changsha, 410008 Hunan China

**Keywords:** Distal humerus fracture, Three-dimensional imaging, Fracture mapping, Heat map, Computed tomography

## Abstract

**Background:**

Distal humerus fractures (DHFs) constitute one-third of elbow fractures approximately. In this study, we aim to define and analyze the fracture lines and morphological features of DHFs using mapping technique.

**Methods:**

One hundred and two DHFs were retrospectively reviewed. All the computed tomography (CT) data were used to manually reconstruct and virtually reduce the DHF fragments to fit a standard 3D model. Smooth curves were depicted accurately onto the surface of the template to represent the fracture lines. All the curves were overlapped onto the model to create the 3D fracture map and heat map.

**Results:**

Our analysis was based on 102 CT images of DHFs, contributed by 59 male and 43 female patients (mean age, 46 years; range, 18-93 years), and included 15 type A, 25 type B, and 62 type C fractures. On mapping, the hot zones were located in the radial fossa, coronoid fossa, olecranon fossa, and the external part of the trochlear. Conversely, the cold zones were noted in medial condyle, the medial side of the trochlear, and the anterolateral area on the supracondylar ridge.

**Conclusions:**

Our study firstly shows the fracture lines and morphological features of distal humeral fractures by three-dimensional mapping technology. Distal humerus fracture lines are characteristic and highly related to the micro-architecture difference of distal humerus, which may provide some guidance for the treatment plan selection and surgical fixation design.

## Introduction

Distal humerus fractures (DHFs) constitute a significant proportion of elbow injuries, which are complex injuries around the elbow caused by high- or low-energy trauma. These fractures are usually attributed to high-energy injuries in younger people and low-energy trauma in elderly female patients, which represent a bimodal age distribution [1].

For DHFs, SOFCOT, AO/OTA, and Dubberley classifications are used to describe fractures and select treatment [2]. As the latest AO/OTA classification which introduced the use of axial CT views to further describe the location of articular depression, is the most commonly used clinical classification method, it divides DHFs into extra-articular (13A), partial articular (13B), and complete articular (13C) types [3]. These types, and their subtypes, describe fracture patterns, stability, and predict prognosis. However, with the development and advancement of radiography, the abovementioned classical classification may have some limitations to give accurate information about the morphology of actual fracture.

Fracture map, first proposed by Armitage et al. [4] in 2009, is a method of superimposing the fracture lines of multiple fracture models on a normal model through CT three-dimensional reconstruction, and visually displaying the shape of this type of fracture. Previous researches have been conducted to investigate the fracture morphology by using 2D fracture map, including tibial plateau fracture [5], pilon fracture [6], and intertrochanteric fracture [7]. However, little work has been done on the distal humeral fracture.

In this study, we aim to define and analyze the fracture lines and morphological features of distal humeral fractures by three-dimensional mapping technology. We hypothesized that this technology would demonstrate precise information about the fracture line distribution. With better understanding of DHFs anatomy, it may aid to develop the fracture classification, treatment plan selection, surgical fixation design, statistics of fracture sites, and the formulation of standardized fracture models.

## Methods

### Subjects

**Fig. 1:**
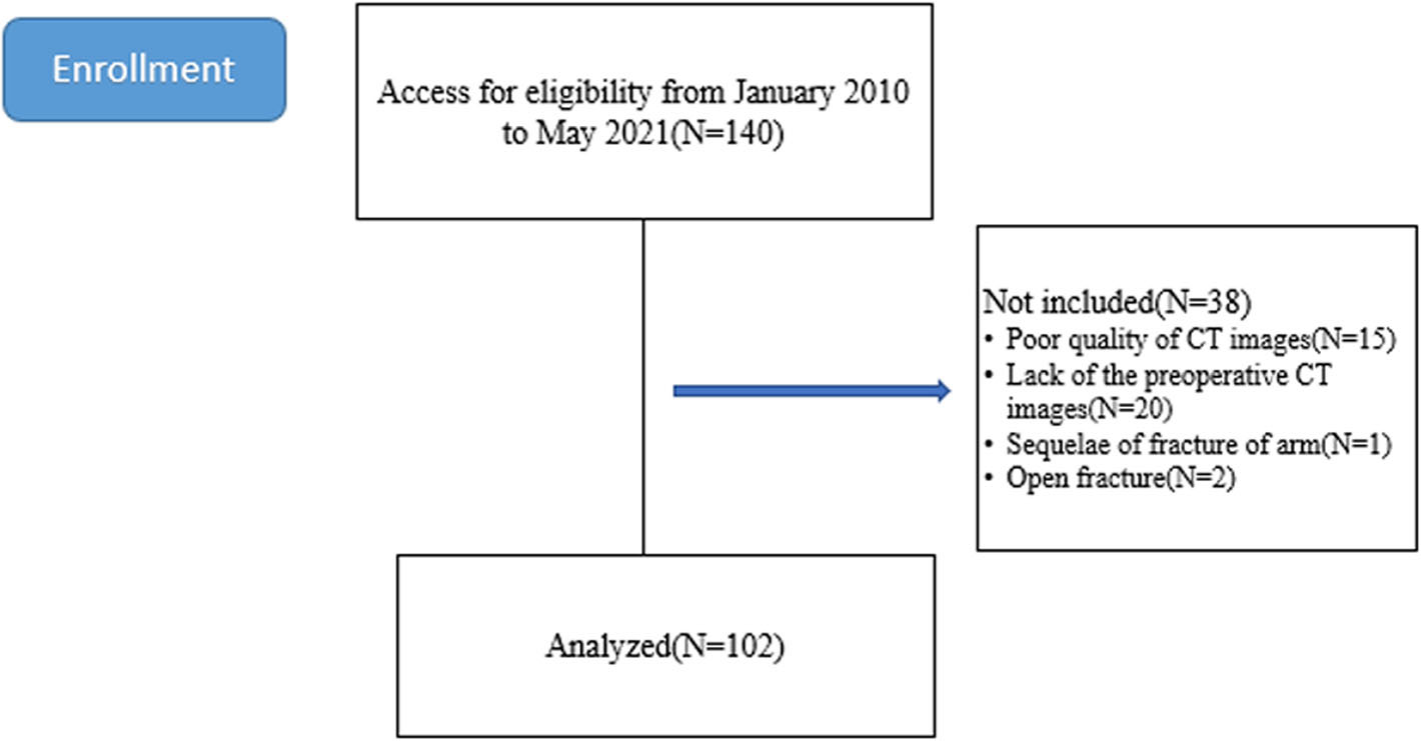
Research flowchart of enrollment

Between January 2010 and May 2021, a total of 140 adult patients with fracture of the distal humerus at the Xiangya Hospital of Central South University, Hunan, China, were enrolled. Exclusion criteria consisted of age < 18 years, CT images with a slice thickness of > 3 mm, open or pathological elbow fractures, developmental dysplasia; heterotopic ossification, or a history of elbow surgery. Fifteen patients were excluded due to poor quality of CT images. Twenty patients were excluded for lacking of the preoperative CT images, one was excluded for sequelae of fracture of arm, and two were excluded because of open fracture. Finally, one hundred and two distal humerus fractures fit the inclusion and exclusion criteria and were included in this study (Fig. 1). There were 59 men and 43 women. The average age was 46 years (range 18–93 years). All fractures were identified using the OTA/AO classification. This retrospective study has been approved by the ethical committee of the Xiangya Hospital of Central South University (No. 202104075).

### Fracture mapping

**Fig. 2:**
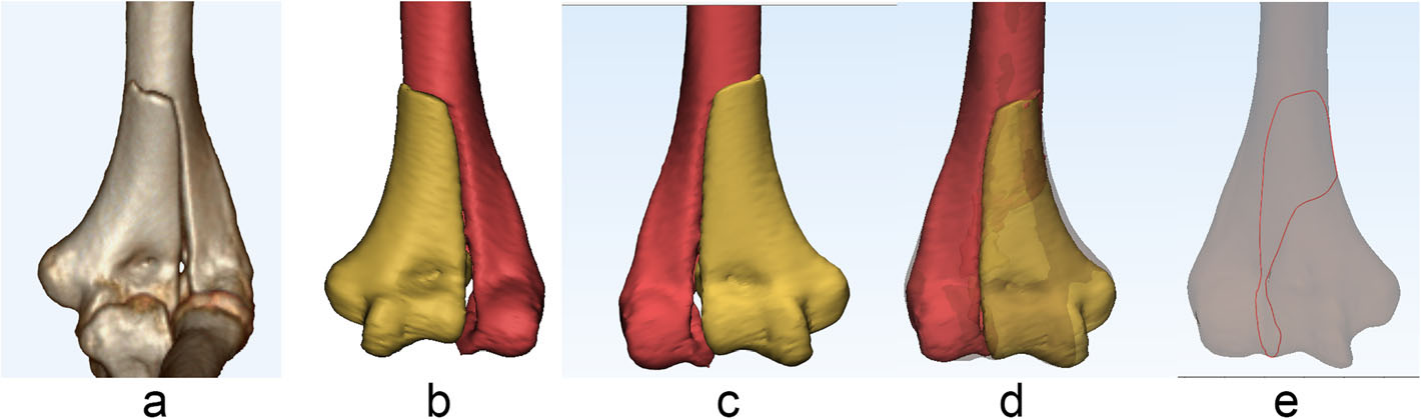
Mapping method of distal humerus fractures. **a** 3D image of distal humerus fracture in CT scans. **b** Major fragments were reconstructed in Mimics. **c** Model of left-sided humerus was mirrored to make the orientation match that of the right-sided humerus in 3-Matic. **d** The reconstructed fragments were reduced, moved, rotated, and normalized to best match the normal model of the distal humerus. **e** Fracture lines were delineated on the template

The 3D fracture maps were produced using previous technique described by Zhang et al. [8]. Original Digital Imaging and Communications in Medicine (DICOM) files of selected CT scans were collected from the Picture Archiving Communications System (PACS) database. A normal distal humerus of a 37-year-old female with no observable elbow pathology was taken as the standard model. Then, the digital imaging and communications in medicine data for all participants were uploaded into Mimics 21.0 software (Materialise, Leuven, Belgium). After we used a threshold of 226 Hounsfield units to manually mark the bone structures of every distal humerus fracture fragment in every axial, sagittal, and coronal slice, the fragments of distal humerus were separated and reconstructed. Then, the reconstructed model was exported to 3-Matic 13.0 software (Materialise, Leuven, Belgium) for further analysis. Reference was made to landmarks, including trochlea, capitellum, coronoid fossa, and olecranon fossa for alignment and standardization. All 57 models of left-sided humerus were mirrored to make the orientation match that of the right-sided humerus. Following it, the reconstructed fragments were moved, rotated, and normalized to best match the normal model of the distal humerus. Then, smooth curves were depicted accurately onto the surface of the 3D template to represent the fracture lines of each DHF in 3-Matic, and fracture maps were made by overlapping all the curves onto the model (Fig. 2). Finally, graphical superimposition of all fracture lines was transferred to E-3D software (Central South University, Changsha, China) to create 3D heat maps. The density of fracture lines was showed in 3D heat maps using colors from deep blue (low density) to red (high density).

### Statistical analysis

Patient characteristics were summarized as the proportion or the mean and standard deviation as well as frequencies and percentages were used for categorical variables. All statistical analyses were performed using IBM SPSS Statistics version 26 (IBM, Armonk, New York, USA). The analysis of fracture morphological mapping was descriptive.

## Result

The patient characteristics are summarized in Table 1. Among the 102 CT images, there are 59 male and 43 female patients, including 57 (56%) left elbow injuries, 45 (44%) right elbow injuries, with a mean age of 46 years (range, 18-93 years). The OTA/AO fracture types are distributed as follows: 15 type A, 25 type B, and 62 type C fractures.

**Table 1:** Patient demographics

Demographic	Data (***n*** = 102)
**Mean age, years (SD)**	
Male	37.1 (16.7)
Female	56.2 (15.0)
Total	45.5 (18.6)
**Sex,*n*(%)**	
Male	59 (57.8)
Female	43 (42.2)
Total	102 (100.0)
**Humerus,*n*(%)**	
Left only	57 (55.9)
Right only	45 (44.1)
Total	102 (100.0)
**OTA/AO classification,*n*(%)**	
13.A	15 (14.7)
13.B	25 (24.5)
13.C	62 (60.8)
Total	102 (100.0)

*SD* standard deviation, *OTA/AO* Orthopaedic Trauma Association/AO Foundation

**Fig. 3:**
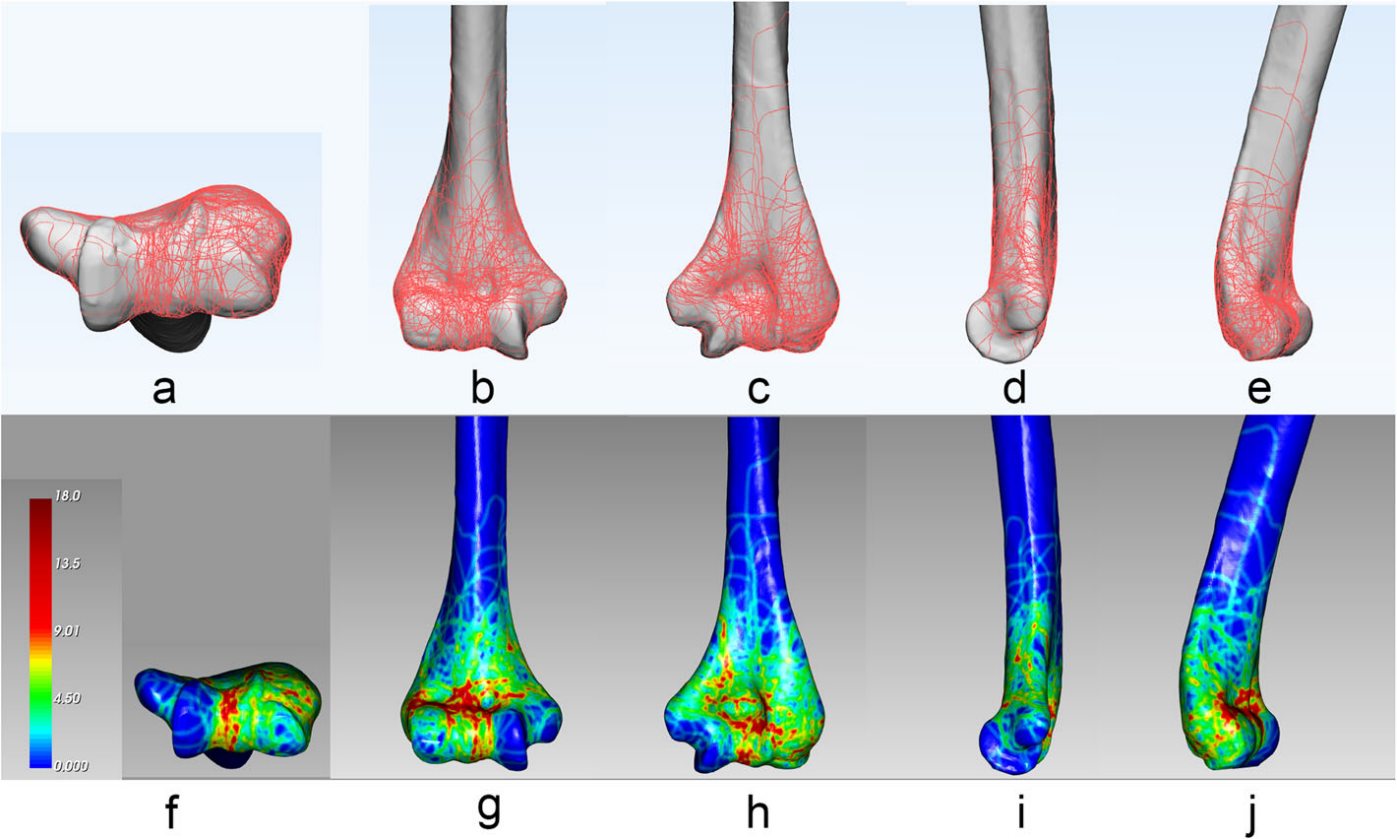
All the fracture lines of 102 patients were overlapped onto the model to create fracture maps in 3-Matic (**a**-**e**) and transferred to E-3D to create 3D heat maps (**f**-**l**), including the axial, anterior, posterior, medial, and lateral views

One hundred and two cases of distal humerus fractures were drawn and then fracture map was obtained (Fig. 3). In order to facilitate intuitive comparison and analysis, the initial fracture map was divided into axial, and surrounding view.

### Axial view

The fracture lines were mainly distributed vertically between the two ridges of the trochlea. At the lateral of the main fracture line—the fracture lines on the capitulum had an oblique or vertical pattern, while the fracture fragments of the medial condylar and the medial side of the trochlea were relatively complete.

### Surrounding view

In the anterior and posterior view, most of humeral infracondylar fracture lines were vertical distribution, while transverse, oblique, or vertical fracture lines accumulated in the trans-epicondylar and supracondylar region. The fracture lines were concentrated on the radial fossa, olecranon fossa, and coronoid fossa. Moreover, the fracture lines on the lateral supracondyle were relatively unconcentrated. In the medial view, the fracture lines were mainly distributed above the medial epicondylar; however, there were almost no fracture lines below it. In contrast, in the lateral view, a major fracture line entered the anterior and upper part of the capitulum and exited the posterior and lower part, while the remaining fracture lines were scattered along the lateral side.

### Heat mapping

On the heat map (Fig. 3), the hot zones were located in the radial fossa, coronoid fossa, olecranon fossa, the lateral side of the capitellum, and the external part of the trochlear. Conversely, the cold zones were noted in medial condyle, the medial side of the trochlear, and the anterolateral area on the supracondylar ridge.

**Fig. 4:**
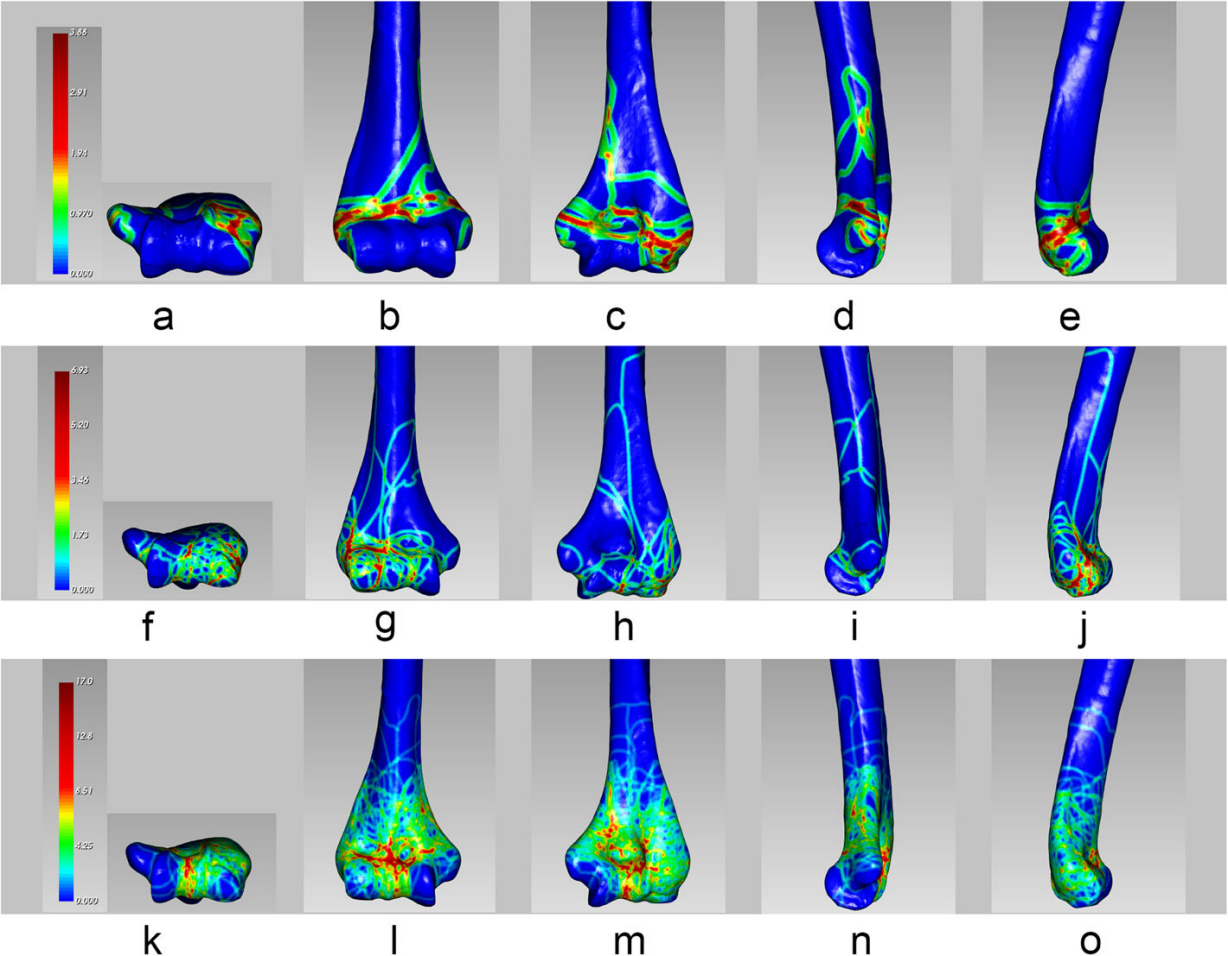
Different fracture maps according to the Orthopaedic Trauma Association/AO Foundation (OTA/AO): type A (**a**-**e**); type B (**f**-**j**); type C (**k**-**o**). Red color represents higher frequency of fracture line density

For OTA/AO type A (Fig. 4), fracture line mainly distributed transversely in the trans-epicondylar region, less in the supracondylar humerus. The rest of the fracture lines could be found in the medial epicondyle and lateral epicondyle of the humerus, that is, around the insertion point of the medial and lateral ligament. For OTA/AO type B, fracture lines were sprawled around the capitellum, and fracture lines on the articular surface were mostly vertically distributed between the capitellum and trochlear. The medial epicondyle of the humerus was rarely involved. For OTA/AO type C, the distribution analyses indicated that the fracture lines in the medial column tended to have higher linear density but more orderly and concentrated distribution fractures compared to those in the lateral column.

## Discussion

With the development of the times and the popularization of medical concepts, intelligent medicine has become a research hotspot [9, 10]. The application of intelligent medical orthopedics is mainly to make the skeleton more structured and standardized through various digital technologies to achieve accurate positioning, so as to meet the personalized needs of patients, and help doctors obtain more satisfactory curative effect and higher work efficiency. At present, the digital technology is based on preoperative CT data for three-dimensional reconstruction of bone, to achieve preoperative three-dimensional measurement of various parameters, so as to complete the digital evaluation of disease. In the preoperative planning, according to the big data or the results of the affected side mirror, the initial orthopedic plan was made. Through three-dimensional visual surgery simulation and finite element analysis, the most accurate orthopedic scheme was obtained. In the future, based on computer vision technology and orthopedic clinical image big data, we may further quantify the skeleton, and import the clinical diagnosis of big data and the diagnosis and treatment scheme of orthopedic experts into the computer system, so as to establish an “intelligent expert system” to serve orthopedic diagnosis and treatment. However, more basic work is needed to realize intelligent expert. Due to the increasing focus on big data analysis, 3D CT-based fracture mapping has been widely used in the field of orthopedics, such as the fracture characterization of proximal femur [8], tibial plateau [11, 12], distal radius [13], or patellar [14], as originally described by Cole et al. [6]. In our study, we have firstly described the fracture lines and morphological features of distal humeral fractures by three-dimensional mapping technology.

It is relatively uncommon of distal humerus fracture in adults, which comprise 2% of all fractures and one-third of elbow fractures approximately. Recent study [15] had divided the patients of distal humerus fracture in two groups based on high or low energy of trauma, and marked differences were found in sex, age, and fracture pattern. Type C fracture among our patients is prominent, and the mean age of female (56) is higher than male (37) in our study, which may indicate that older women are at particularly high risk of fracture, with multiple fracture fragments and poor bone quality often complicating surgical treatment.

Marcoin et al. [16] have researched the relationship between the distal humeral bone density and supracondylar fracture threshold. By testing 21 cadaveric distal humeri, they find a correlation between bone mineral density and the fracture threshold (*r* = 0.7321). In the previous study conducted by Diederichs et al. [17], the distribution of cancellous bone and cortical thickness in the distal humerus were quantitatively evaluated by pQCT, which demonstrated total bone mineral density decreased continuously from the distal diaphysis to the trochlea, suggesting that low bone density may predispose to fractures or comminution. These findings were similar to those displayed on our fracture map and heat map, which showed a higher fracture frequency in the metaphyseal regions than diaphyseal regions. Another study [18] has demonstrated that, in the distal humeral section, the anterior section of the lateral condyle has the greatest bone volume and the posterior part has the smallest bone volume. Cortical thickness in the trans-epicondyle area is the thickest in the posterior medial, the thinnest in the anterior aspect, and the posterior lateral aspect followed, which may be a well explanation of the cold zone observed around the medial condyle and the relatively high fracture frequencies around the lateral condyle. According to our study, the distribution of fracture lines was highly related to the micro-architecture difference of distal humerus. The correlation between our fracture maps and distal humerus osseous micro-architecture analysis may help to improve treatment plan selection or surgical fixation design to better treat the distal humerus injuries. For example, the differences in bone density and the frequency of fractures in the diaphyseal and metaphyseal regions of the humerus can help guide the design of different plates and the clinician’s surgical strategies for addressing this issue, such as screw placement or plate selection. Furthermore, the correlation also indicates that, for the plate fixed in the posterolateral aspect of the lateral condyle (the relatively prone site of fracture), there may be a potential weakness [18].

**Fig. 5:**
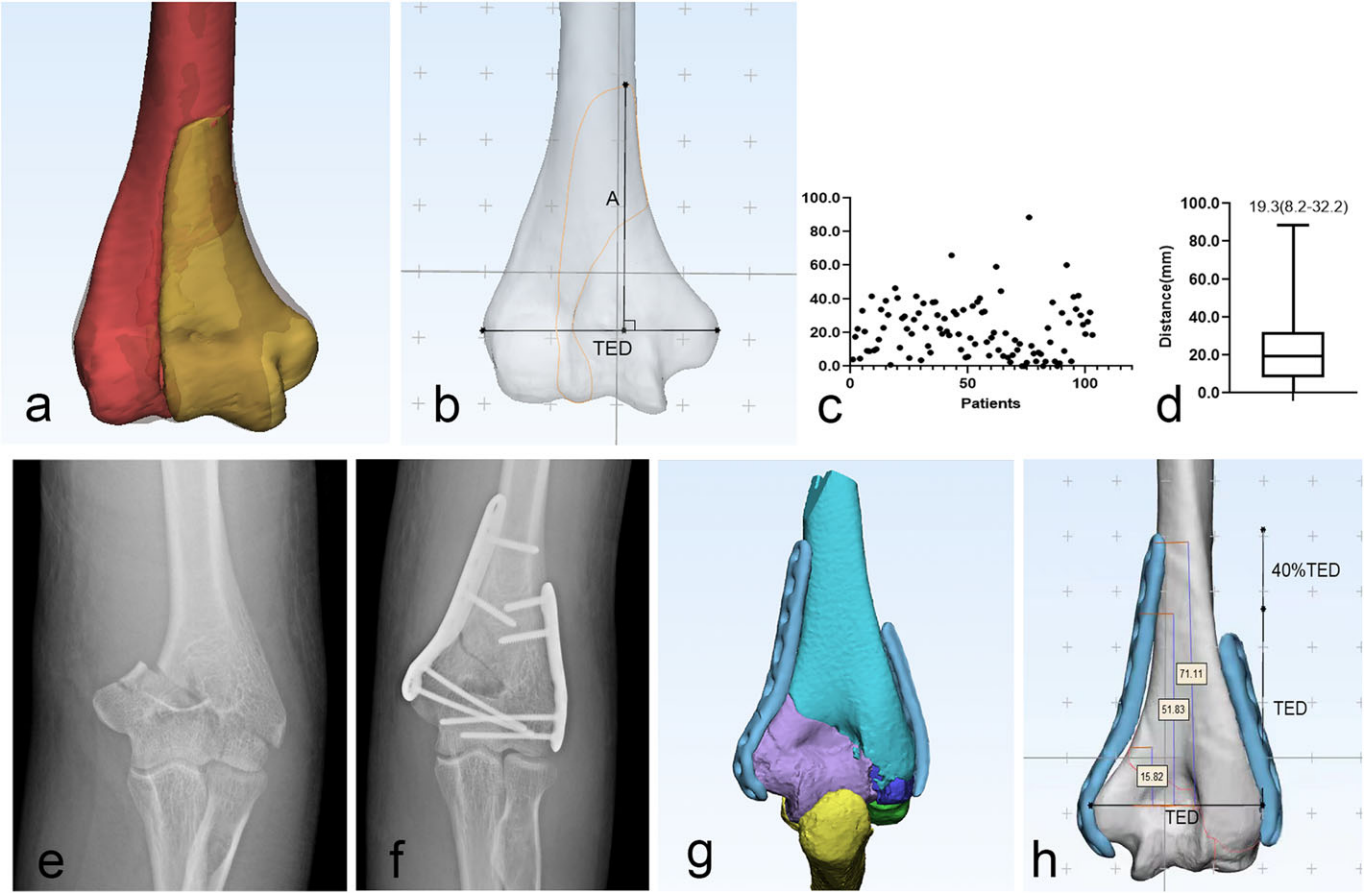
**a**-**b** Measurement of the distance between the most proximal point of fracture lines to the trans-epicondylar line. A, distance between the most proximal point of fracture lines to the trans-epicondylar line. TED, trans-epicondylar distance. **c**-**d** The scatter plot and box plot of all the distance of 102 patients. **e**-**h** Measurement of the length of the plate when the screws were placed in the two or four proximal holes near the fracture line

Distal humeral fractures can be fixed by a variety of surgical approaches, such as anterior, anterolateral, posterior, and modified posterior. In each of these approaches, however, one of the major concerns is injuring the radial nerve (RN), which passes diagonally through the spiral groove from the medial to the lateral side of the posterior surface of the humerus [19]. During surgical interventions, such as external fixator pin placement, transfixing wires (e.g., Ilizarov) or plating, secondary or iatrogenic palsies are not uncommon [20]. Thus, it is vital to identifying of the RN during surgery, especially in the posterior approach. After dissecting 70 fresh cadaveric upper limbs, Kamineni et al. [21] have introduced a safe parameter to decrease the risk to the RN. They reported that on each specimen a positive Pearson correlation coefficient was found between the trans-epicondylar distance (TED) and the radial nerve lateral height distance(*r* = +0.95), and the length of radial nerve lateral height was 1.4 to 2.0 times the TED. Based on these, they concluded that 100% TED is safe and an extra 40% TED could be used as a moderate safe area [22]. In our study, we have measured the TED of the standard model and all the distance between the most proximal point of fracture lines to the trans-epicondylar line (Fig. 5). The TED of the standard model was 53.4 mm. We found that only four fracture lines were out of 100% TED, three were in the extra 40% TED region (59.0, 60.0, 65.9 mm), and one was in the area of 140-200% TED (88.5 mm). For the remaining fracture lines, for example (Fig. 5e, f), when the Acumed plate was placed on the fracture model and the screws were placed in the two proximal holes near the fracture line, the plate was approximately 51.83 mm in the 100% TED range. When the screws were inserted into the proximal four holes, the plate was approximately 71.11 mm, within an additional 40% TED range. However, as same as treating distal shaft humeral fractures, the dorsal flat surface is suitable for plating and we can put the plate more proximally to increase the proximal fixation on distal humeral fractures, but attention still remains to the risk of injury to the radial nerve.

In the analysis of findings, the limitations of this study should be acknowledged.

First, the sample size of this study is small. Some conservative-treated patients without CT scan were not included. As a level I trauma center in our region, patients are usually sent to our hospital for treatment after a CT examination in another hospital, thus preoperative CT data are often not available in our hospital system. Besides, compared to community hospitals, patients in our study often have higher rates of complex distal humerus fracture, which may lead to some bias. Second, subjective process to draw the fracture line and qualitative assessment evaluate fracture maps are the other limitations. Third, the injury mechanism of distal humerus fractures and its relationship with fracture maps are not analyzed in detail. Fourth, the patella of a 37-year-old healthy female patient’s right elbow was used as a template. The average age of the patients in our study group was 46 years old and 59 (57.8%) were male. Elderly male patient without pathology is preferred due to slight changes in humeral morphology in the normal population. Further study could increase the sample size and investigate the difference of distal humerus fracture map combined with injury mechanism, different age groups, sex, and fracture pattern.

In conclusion, to our knowledge, our study is the first study revealing the fracture lines and morphological features of distal humeral fractures by three-dimensional mapping technology. We believe it could aid to develop the fracture classification, treatment plan selection, surgical fixation design, statistics of fracture sites, the formulation of standardized fracture models, and help clinicians better understanding DHFs anatomy.

## Data Availability

All the data are available if qualified authors apply for them.

## Abbreviations

DHFs: Distal humerus fractures
CT: Computed tomography
TED: Trans-epicondylar distance
